# Transcriptome Profiling of Peripheral Blood Cells Identifies Potential Biomarkers for Doxorubicin Cardiotoxicity in a Rat Model

**DOI:** 10.1371/journal.pone.0048398

**Published:** 2012-11-27

**Authors:** Valentina K. Todorova, Marjorie L. Beggs, Robert R. Delongchamp, Ishwori Dhakal, Issam Makhoul, Jeanne Y. Wei, V. Suzanne Klimberg

**Affiliations:** 1 Department of Surgery/Breast Surgical Oncology, University of Arkansas for Medical Sciences, Little Rock, Arkansas, United States of America; 2 Central Arkansas Veterans Healthcare System, Little Rock, Arkansas, United States of America; 3 Department of Medical Genetics, University of Arkansas for Medical Sciences, Little Rock, Arkansas, United States of America; 4 Department of Epidemiology, University of Arkansas for Medical Sciences, Little Rock, Arkansas, United States of America; 5 Department of Hematology/Oncology/Hematology, University of Arkansas for Medical Sciences, Little Rock, Arkansas, United States of America; 6 Department of Geriatrics, University of Arkansas for Medical Sciences, Little Rock, Arkansas, United States of America; Morehouse School of Medicine, United States of America

## Abstract

**Aims:**

Doxorubicin (DOX), a widely used anticancer agent, can cause an unpredictable cardiac toxicity which remains a major limitation in cancer chemotherapy. There is a need for noninvasive, sensitive and specific biomarkers which will allow identifying patients at risk for DOX-induced cardiotoxicity to prevent permanent cardiac damage. The aim of this study was to investigate whether the expression of specific genes in the peripheral blood can be used as surrogate marker(s) for DOX-induced cardiotoxicity.

**Methods/Results:**

Rats were treated with a single dose of DOX similar to one single dose that is often administered in humans. The cardiac and peripheral blood mononuclear cells (PBMCs) genome-wide expression profiling were examined using Illumina microarrays. The results showed 4,409 differentially regulated genes (DRG) in the hearts and 4,120 DRG in PBMC. Of these 2411 genes were similarly DRG (SDRG) in both the heart and PBMC. Pathway analysis of the three datasets of DRG using Gene Ontology (GO) enrichment analysis and Ingenuity Pathways Analysis (IPA) showed that most of the genes in these datasets fell into pathways related to oxidative stress response and protein ubiquination. IPA search for potential eligible biomarkers for cardiovascular disease within the SDRG list revealed 188 molecules.

**Conclusions:**

We report the first in-depth comparison of DOX-induced global gene expression profiles of hearts and PBMCs. The high similarity between the gene expression profiles of the heart and PBMC induced by DOX indicates that the PBMC transcriptome may serve as a surrogate marker of DOX-induced cardiotoxicity. Future directions of this research will include analysis of PBMC expression profiles of cancer patients treated with DOX-based chemotherapy to identify the cardiotoxicity risk, predict DOX-treatment response and ultimately to allow individualized anti-cancer therapy.

## Introduction

Doxorubicin (DOX, Adriamycin) is the key component of many cytotoxic regimens for treatment of different adult and childhood cancers [1]. DOX cardiotoxicity remains a major limitation in cancer chemotherapy [2]. The cardiac events occurring in cancer patients treated with DOX-based regimens have been estimated to be between 4% and 45% [3], [4], [5] and the frequency of subclinical cardiotoxicity in children treated with DOX was estimated to be up to 57% [6]. DOX-induced cardiotoxicity can develop during the therapy or in the months after its completion (early or acute cardiotoxicity) or it can manifest itself several years after the treatment (late onset chronic) cardiotoxicity) [7],[8],[9]. DOX-associated cardiac toxicity results from a cumulative dose-related effect [10],[11]. The most commonly used DOX dose schedule is 60 to 75 mg/m^2^ in each intravenous injection administered at 21 to 28-day intervals and each administration constitutes additive or sequential irreversible cardiac damage [12].To date however, no biomarker for early pre-symptomatic detection of DOX cardiotoxicity has been validated [13]. Currently, the clinical methods used for detection of pre-symptomatic DOX-induced cardiotoxicity [assessment of left ventricular volume (LVV) and ejection fraction (LVEF) using echocardiography and radionuclide angiography] show low diagnostic sensitivity and low predictive power, and identify the existing cardiomyopathy rather than prevent it [14],[15]. Invasive techniques such as endomyocardial biopsy cannot be performed routinely [16]. Blood cardiac biomarkers such as cardiac troponins [17] and cardiac natriuretic peptides [18] have been evaluated in animal models and in clinical studies [19] but their diagnostic and prognostic values in humans have not been validated [20].

Several reports indicate that there is a considerable variation in an individual's susceptibility to the cardiotoxic effects of DOX [21]. A retrospective analysis of three clinical trials indicates that DOX cardiomyopathy can occur at low doses, suggesting the presence of increased phenotypic sensitivity in some individuals [22]. For example, DOX doses >1000 mg/m^2^ were tolerated by some patients, whereas others developed DOX-induced cardiotoxicity after <200 mg/m^2^ [23]. The presence of such widely varying sensitivity suggests that early identification of patients who may be at risk for DOX-induced cardiac damage might help clinicians to reduce the incidences of cardiotoxicity and the associated morbidity [24].

Because of their ease of accessibility, the expression profiles in “surrogate” tissues, such as peripheral blood mononuclear cells (PBMCs), are of interest for determining whether their gene expression patterns may predict clinical outcomes in various diseases. A number of studies have demonstrated that transcriptomic changes in peripheral blood can serve as biomarkers of exposure to xenobiotics or as biomarkers of pathological changes occurring in other tissues [25], [26] and [27]. The continuous interactions between blood cells and the entire body, combined with the fast turnover rate of blood cells, gives rise to the possibility that subtle changes occurring in association with injury or disease within the cells and tissues of the body may trigger specific changes in gene expression of blood cells. A high correlation between the gene expression profiles of other tissues and the corresponding PBMC has been reported [28].

The present study aimed to compare the gene expression profiles of hearts and PBMCs of rats treated with a single dose of DOX similar to the single dose administrated to humans with the ultimate goal to identify biomarkers for early prediction of DOX cardiotoxicity in “surrogate” more easily obtainable tissues such as the peripheral blood.

## Materials and Methods

### Animals and treatment

This study was carried out in strict accordance with the recommendations in the Guide for the Care and Use of Laboratory Animals of the National Institutes of Health. The protocol was approved by the Animal Care and Use Committee at the Central Arkansas Veterans Healthcare System (CAVHS) (Protocol # 9-11-3), where the animals were housed, treated and sacrificed. A total of 17 female Sprague-Dawley rats were used (Harlan Sprague-Dawley, Inc.,Indianapolis, IN). The rats were maintained in standard cages (two animals per cage) in the Animal Care Facility of CAVHS (Little Rock, AR, USA). We have used female rats in this study, as it has been reported that androgens play a protective role against the development of DOX-induced cardiotoxicity [29].The rats were subjected to a 12-hour dark/light cycle and food (Harlan Teklad, Madison, WI), and water were provided ad libitum. The rats were randomized into 2 groups: experimental group (n = 9) and control group (n = 8). The rats in the experimental group were injected intraperitoneally (i.p.) with a single dose of 12 mg/kg DOX, as DOX hydrochloride (Sigma Chemical Co., St. Louis, MO, USA) diluted in saline. This dose of DOX, depending on the body weight of the rat is approximately similar to 65–75 mg/m^2^ for humans [30]. The rats were injected intraperitoneally (i.p.) with a 2 mg/kg DOX-saline solution with the volume calculated for the individual rat's body weight. The i.p. route of administration of DOX in small animals is the standard route of DOX administration [31]. In the clinical practice DOX concentrate for infusion is usually 2 mg/ml doxorubicin hydrochloride in saline (0.9% Sodium Chloride), “given via the tubing of a freely running intravenous infusion” [32]. The control rats were injected i.p. with 0.1 ml saline so that they were exposed to the same stress. All rats were sacrificed under deep isoflurane anesthesia 48 hours after DOX administration.

### Sample collection, biochemical assays

At sacrifice whole blood (2.5–3 ml) was collected via heart puncture into K_2_EDTA-containing tubes. Samples from the left ventricle (LV) of the heart were collected, snap-frozen in liquid nitrogen, and stored at −80°C until used. PBMCs were separated from the whole blood diluted 1∶2 with PBS by gradient centrifugation using Ficoll Paque Plus (GE Healthcare Biosciences, Upsala, Sweden).

The fluorescent properties of DOX were used to verify the equal concentrations of DOX in the blood and heart samples [33]. The fluorescent levels were expressed as µg/mg protein or µg/ml blood. Protein content of the tissue samples was measured using the Bio-Rad Protein Assay (Bio-Rad Laboratories, Hercules, CA).

Complete leukocyte counts and differentials were obtained using an automated hemocytometer (Hemavet 950FS Veterinary System, Drew Scientific Group; Oxford, CT) according to the manufacturer's instructions.

Blood chemistry values were determined in 5 control rats and 6 rats treated with DOX using VetScan VS2 analyzer (Abaxis, Inc., Union City, CA). The Comprehensive Diagnostic Profile reagent rotor was selected for quantitative determination of 14 parameters, including alanine aminotransferase (ALT), albumin (ALB), alkaline phosphatase (ALP), amylase (AMY) total calcium (CA++), creatinine (CRE), globulin (GLOB), glucose (GLU), phosphorus (PHOS), potassium (K+), sodium (NA+), total bilirubin (TBIL), total protein (TP), and urea nitrogen (BUN).

Statistical analyses of the biochemical assays results were performed using ANOVA (StatView II) and the results were expressed as mean ± standard error (SE). P<0.05 was considered to indicate a statistically significant difference.

### RNA extraction, microarray and data analysis

RNA was extracted from the PBMC and heart samples from identical rats and numbered accordingly so that samples from the same rat could be identified (AllPrep DNA/RNA/protein mini kit, Qiagen, Valencia, CA) and only samples with a RIN score>7 were used for expression analysis. The RNA integrity number (RIN score) was assessed on a Bioanalyzer (Agilent, Palo Alto, CA, USA) prior to labeling. For each individual sample, 10 ng of total RNA was amplified using Ovation PicoSL WTA system V2 (NuGen Technol., San Carlos, CA). A total of 34 separate samples (17 heart samples and 17 PBMC samples) were used. This allowed us to evaluate sample to sample variability in the gene expression both within and across each group. Each individual heart RNA sample and PBMC RNA sample was amplified to have sufficient quantities of RNA to proceed with both the arrays and QPCR. The Ovation Pico WTA System is a whole transcriptome RNA Amplification System based on Ribo-SPIA® technology, initiating at both the 3′ end and randomly throughout the entire transcriptome. Total RNA (500 pg) from each of the 34 samples was used to generate amplified cDNA. The amplified cDNA was cleaned with the MinElute Reaction Cleanup Kit and labeled using Encore Biotin IL Module (Nugen Technol, San Carlos, CA.). The labeled cDNAs (3 µg) were hybridized to Illumina RatRef-12 Expression BeadChips. Each array is comprised of 22,523 probes selected primarily from the NCBI RefSeq database (Illumina, San Diego, CA). Recently published papers [34], [35] did not report duplicated or triplicated arrays. However, we run duplicated samples from 7 randomly selected rats (4 DOX-treated and 3 controls), i.e 14 samples, including 7 heart- and 7 PBMC samples from matching rats in order to verify our results. Following hybridization all forty-eight arrays were washed, labeled with Cy3 streptavidin, washed again to remove unincorporated Cy3 and quickly dried. The arrays were scanned on the Illumina iScan Reader and the data were imported into Illumina GenomeStudio software to assess quality metrics of each sample and Illumina internal controls (sample independent and sample dependent) which are included on each array. The raw data without any background subtraction or analysis were exported to an excel table for analysis using Statistical Analysis System (SAS).

Intensity data from microarrays were analyzed as follows: 1) raw probe intensities were log2 transformed, 2) low intensities (background –level intensities) were filtered out, 3) data from each array were then normalized by a median subtraction, 4) comparisons (control vs. DOX-treated) were performed using a moderated Student's t-test per probe (shrinkage-style test statistic), and 5) significance was evaluated by the false discovery rate [36]. More specifically, probes were classified as expressed or not expressed (background –level intensities) based on their average log-intensity over arrays [37] and [38]. Within each array, the median of the log2-transformed intensities computed from the set of ‘not expressed’ probes was subtracted from the log2 intensities to normalize the data. The moderated Student's t-test is discussed by Wright and Simon [39]. For the expressed probes, p-values from the moderated t-test were further adjusted using the null distribution of the ‘not expressed genes’ (motivated by [40], explained further in [37]). Statistical significance was set at false discovery rate <0.05 (FDR, an estimate of the false-positive proportion of the expressed genes that are claimed to be differentially regulated) [41]. Only statistically significant genes with greater than 2-fold change (FC) in expression between groups were retained.

### Gene ontology (GO) enrichment analysis

The gene symbols, which Illumina had assigned to the array probes, were matched with gene ontology (GO) terms using gene annotations for the rat (www.geneontology.org, downloaded on 2/19/2012). GO terms enrichment analysis of the genes that were significantly expressed in both tissues was performed in terms of biological process, molecular function and cellular component. For the 2,400 GO terms with more than 4 expressed genes on the array, we computed an overall probability that the associated set of expressed genes were unaffected by DOX treatment [42], [43]. A false discovery rate was computed for the 2400 analyses within each tissue to provide an adjustment for multiple testing [44]. All computations were programmed using Matlab (www.mathworks.com); in particular the bioinformatics toolbox and the statistics toolbox were used.

### Pathway Analysis

The Ingenuity Pathways Analysis (IPA, Ingenuity® Systems, http://www.ingenuity.com) software was used to identify the significantly enriched canonical pathways and to build, and analyze the significantly enriched molecular interaction networks from the lists of DRG. The canonical pathways were identified based on the IPA library of canonical pathways that were most significant to the data set. Genes from the data set that were associated with a canonical pathway in IPA were then considered for the analysis. The significance of the association between the data set and the canonical pathway was measured in two ways: 1) a ratio of the number of genes from the data set that map to the pathway divided by the total number of genes that map to the canonical pathway is displayed. 2) Fisher's exact test was used to calculate a P-value determining the probability that the association between the genes in the dataset and the canonical pathway may be explained by chance alone. Networks were ranked by a score: the higher the score, the lower the probability of finding the observed data set of genes in a given network by chance. The score takes into account the number of dataset genes and the size of the network and is the negative log of the *P*-value. The Functional Analysis of a network identified the biological functions and/or diseases that were most significant to the genes in the network, as well as the potential toxicity and safety of compounds, associated with a given dataset.

### Real time QPCR

QPCR was used for evaluation and confirmation of the gene expression data. cDNAs amplified using Nugen Ovation Pico WTA system were used. All specific primers (Taqman Gene Expression Assays), including the internal control (eukaryotic 18S rRNA) were purchased from Applied Biosystems (Foster City, CA). Prior to ordering the primers, all probes from the Illumina array were blasted on the NCBI website to verify that they correctly matched the gene defined. QPCR was performed using the ABI 7900HT standard amplification protocol with each specific target using Taqman Universal Fast PCR master mix (Applied Biosystems, Foster City, CA) in a 10 µl PCR reaction (0.5 µl primer/0.5 µl H_2_O/5 µl master mix), according to the manufacturer's protocol. The individual samples were assayed in triplicate on a 96 well plate. Data were analyzed using the 2-deltadelta CT method [45], which is used to compare the changes in gene expression. Delta delta CT is the difference in threshold cycles for the target and control samples. The Ct values (the cycle number at which detectable signal is achieved) of both the control and the samples of interest are normalized to an appropriate endogenous housekeeping gene, which in our studies was 18S. The formula used was:
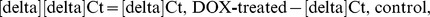
where [delta]Ct,DOX-treated is the Ct value for any DOX-treated sample normalized to the endogenous housekeeping gene; and [delta]Ct control is the Ct value for the calibrator also normalized to the endogenous housekeeping gene.

## Results

### Biochemical parameters

**Figure 1:**
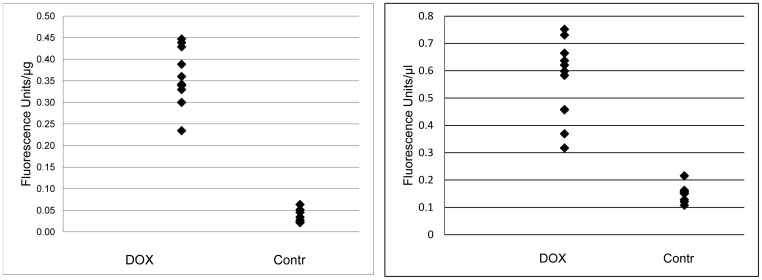
DOX concentration in hearts (A) and blood (B) of rats 48 hours following administration of 12 mg/kg DOX. *P<0.01*.

DOX concentrations in the blood and hearts were measured in order to verify the equal treatment conditions for all rats in the experimental group. The results from the analysis of DOX concentration showed that 48 hours after DOX administration the average concentration of DOX in the hearts was 0.342±0.22 µg/mg protein in the experimental groups versus 0.014±0.003 in the controls, p = 0.051 and in the blood 0.591±0.041 µg/ml in the experimental groups versus 0.431±0.071 µg/ml in the controls, p<0.01 (Fig. 1).

**Figure 2:**
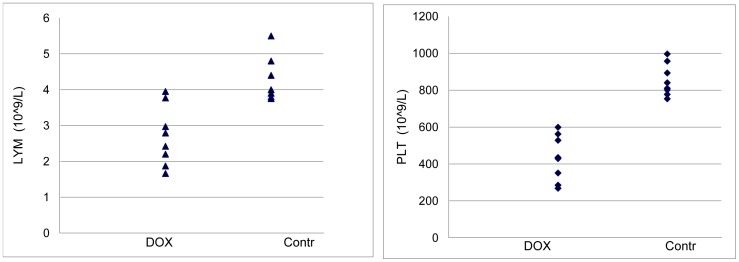
Lymphocytopenia (A) and thrombocytopenia (B) in rats 48 hours after administration of 12 mg/kg DOX. *P*<0.05.

Red blood cell counts, hematocrit, and hemoglobin concentrations were not affected significantly (not shown). The analysis of the blood cell count showed the presence of lymphocytopenia and thrombocytopenia in the rats treated with DOX (Fig. 2). The results from the blood chemistry analysis (Table 1) showed that 6 of the 14 parameters examined were affected by DOX administration, including ALB, ALT, TBIL, CRE, K+ and Globin.

**Table 1:** Blood chemistry values of 5 control rats and 6 rats treated with DOX.

	Controls (n = 5)	DOX-treated rats (n = 6					
	Average	1	2	3	4	5	6
ALB (g/dL)	3.7–4.7	0	3.1	2.9	0	0	0
ALT (U/L)	61–74	149	137	157	204	225	178
TBIL (mg/dL)	0.2–0.4	high	high	high	high	high	high
CRE (mg/dL)	0.2–0.5	high	high	high	high	high	high
K+ (mM)	7.2–9	high	high	high	high	high	high
Globin (g/dL)	1.6–2.5	0	1.4	1.4	0	0	0

### Gene Expression Arrays

The amplified RNA isolated from each sample was used for both gene expression analysis and QPCR. Illumina beadchip gene expression technology was used to identify and compare the early changes in cardiac and PBMC gene expression induced by DOX in rats. The gene expression of LV of the heart and PBMC in individual rats were assayed 48 h after i.p. administration of 12 mg/kg DOX.

**Figure 3:**
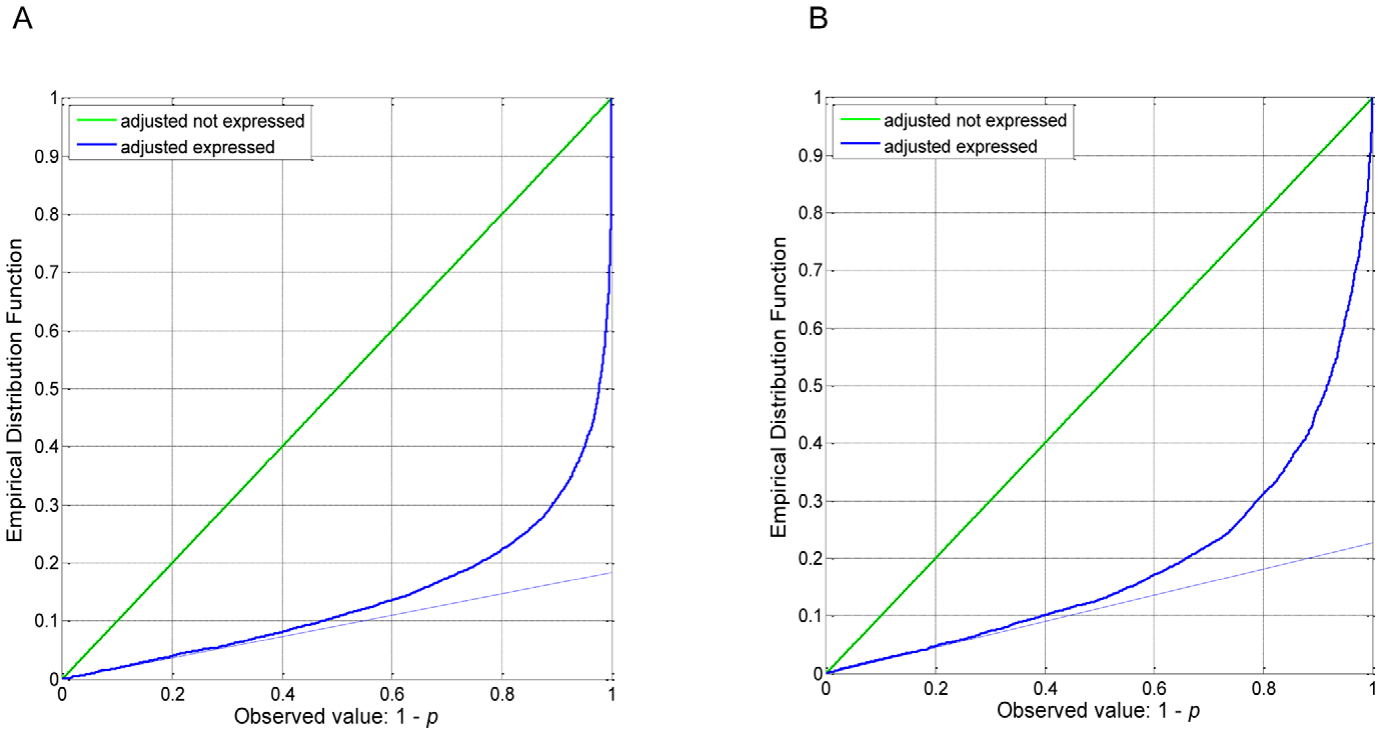
P-value plot for the null hypothesis of no difference in gene expression between heart tissues from DOX treated rats versus heart tissues from control rats (A) and PBMC tissues from DOX treated rats versus PBMC tissues from control rats (B).

The Illumina Rat Ref-12 BeadChip assays contain 22,523 probes. The log2-transformed intensities were averaged for the 34 samples, including heart samples from 17 rats and PBMC samples from the same 17 rats. All samples, independent and Illumina dependent beadchip controls were very good. The duplicated arrays gave identical results. Probes not associated with a known gene were not analyzed further. Some genes had multiple probes and only the probes with the largest average expression were retained. With these deletions, there were transcript assays for 21,567 genes. Gene transcripts with average log2-intensities greater than 7.2 (9,145) were considered to be expressed. The effect of DOX treatment was tested using a moderated t-test and the resulting p-values were adjusted to the empirical distribution of the p-values from the genes that were not expressed. The resulting p-value plots of the comparison of DOX treatment versus controls indicated that about 80% of the expressed genes were affected (Figure 3A: π_0_ = 0.1837 in the heart and Figure 3B: π_0_ = 0.2278 in the PBMC [46] and [47].

**Figure 4:**
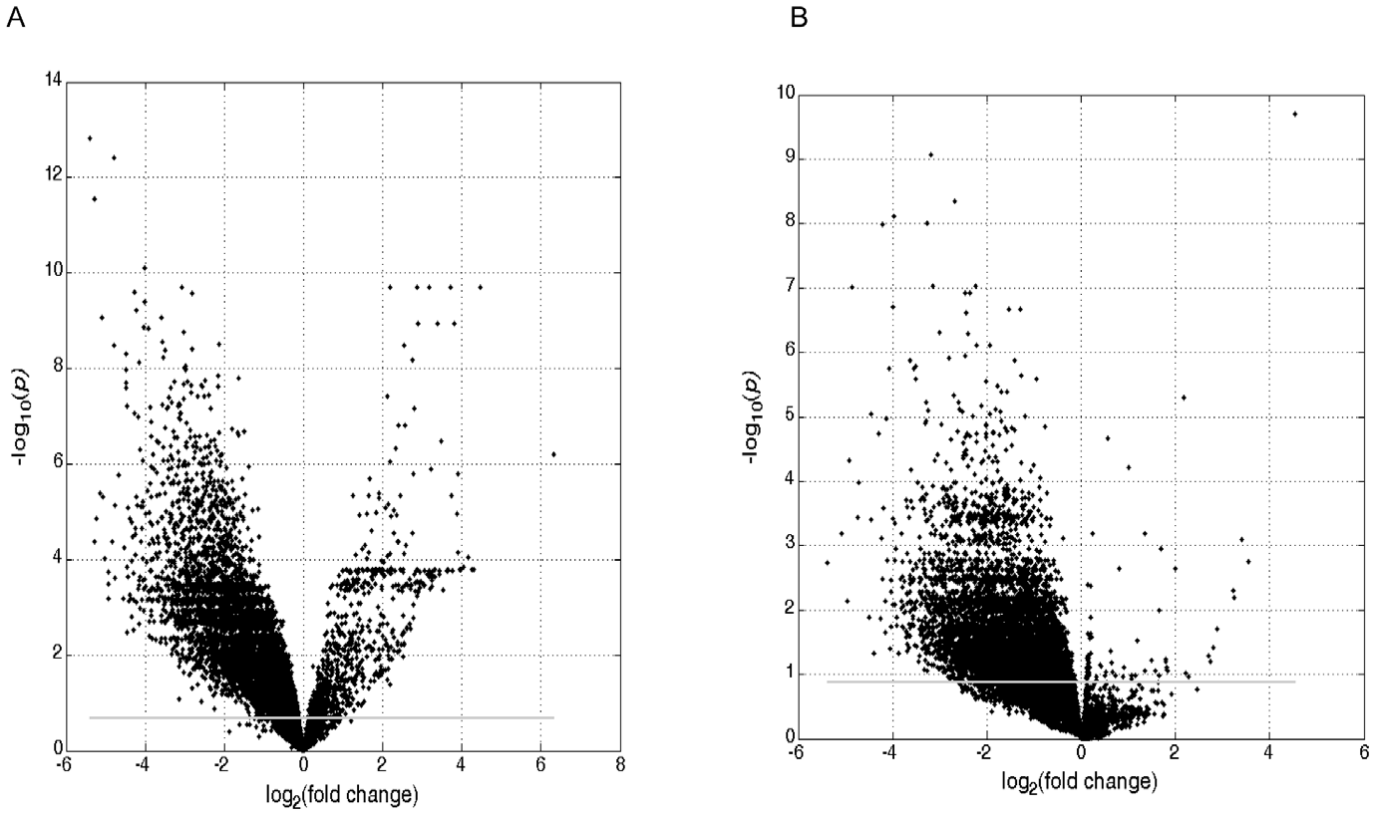
Volcano plot for difference in gene expression between heart tissues from DOX treated rats versus heart tissues from control rats (A) and PBMC tissues from DOX treated rats versus PBMC tissues from control rats (B). The gray horizontal line demarks FDR = 0.05 so values with FDR<0.05 are above the line. FDR>2 have log_2_ fold change >1 and fold change <1/2 have log_2_ fold change <−1.

**Figure 5:**
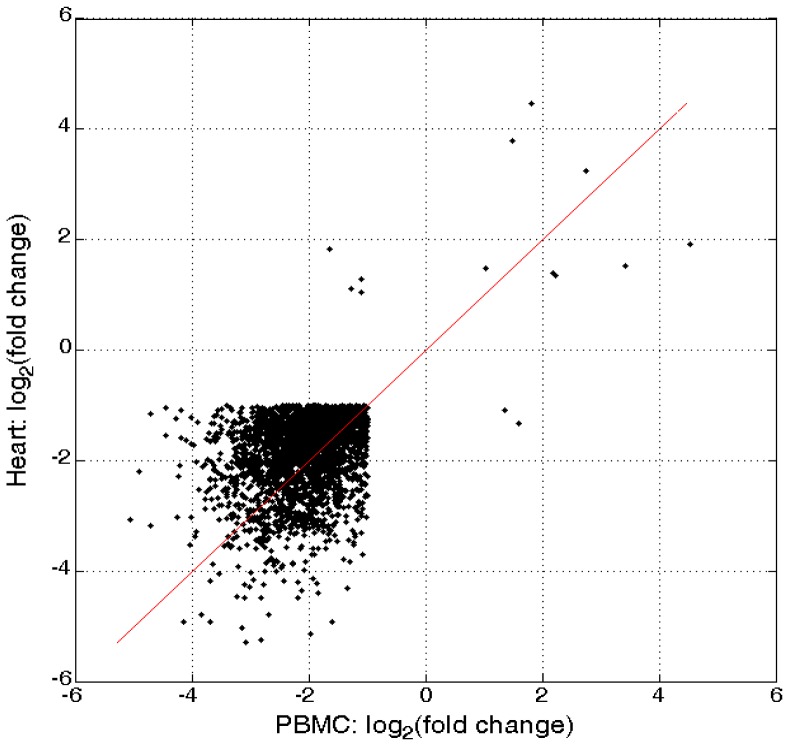
Estimated log2 fold change for the 2,411 SDRG (26.4% of those expressed) that were identified as significant in both heart and PBMC tissues. DOX treatment down-regulated most gene expression with down-regulation stronger in PBMC.

The statistical test of DOX treatment resulted in 4,409 (48.2%) of the expressed genes being identified as significant (FDR<0.05 and fold change >2) in the heart tissue; 382 up-regulated and 4,027 down-regulated. In Figure 4A (volcano plot) the log_2_ fold change (DOX vs control) for the 9,145 expressed genes in the heart is plotted against −log_10_ FDR. The statistical test of DOX treatment resulted in 4,120 (45.1%) of the expressed genes being identified as statistically significant (FDR<0.05) in the PBMC tissue; 31 up-regulated and 4,089 down-regulated. Figure 4B gives the volcano plot for the 9,145 expressed probes in the PBMC. Figure 5 plots the estimated log2 fold change for the 2,411 SDRG (26.4% of the expressed genes), 12 upregulated and 2,399 downregulated that were identified as significant in both tissues. The lists of DOX-induced DRG are presented in Table S1.

All array results may be found at the Gene Expression Omnibus Web site (http://www.ncbi.nlm.nih.gov/geo/) under accession number GSE37260.

### Gene ontology (GO) enrichment analysis

The GO enrichment analysis, which describes genes in terms of their associated biological processes, molecular function and cellular component, found more than 1000 significant GO terms for SRDG at FDR<0.05 (Table S2). The biological processes related to enriched GO categories (n = 1,110) included positive regulation of the following pathways: cell migration; heart, kidney and mammary gland development; angiogenesis; ion transport; skeletal system and muscle organ development; calcium homeostasis and metabolism; regulation of MAPK-, BMP, SMAD-, Wnt-, and Rho protein signaling; wound healing; glycogen biosynthesis; blood vessel morphogenesis; lipid metabolism; regulation of mitotic cell cycle; protein ubiquitination; immune response and multiple others, all highly significant, indicating that DOX cardiotoxicity and DOX cytotoxicity are both multifactorial processes. The most significantly enriched GO terms related to the molecular functions (n = 368) included receptor binding; zinc, calcium, E-box and metal ions binding; DNA and RNA binding, ion transport and activity, ATPase activity and others highly significantly affected. Some of the most significantly affected cellular components (n = 225) were cytoplasm, basolateral plasma membrane, nucleus, synapse, nucleolus, intracellular, cell junction, synaptic vesicle membrane and extracellular region, sarcoplasmic reticulum and desmosome.

### Pathways Analysis

**Figure 6:**
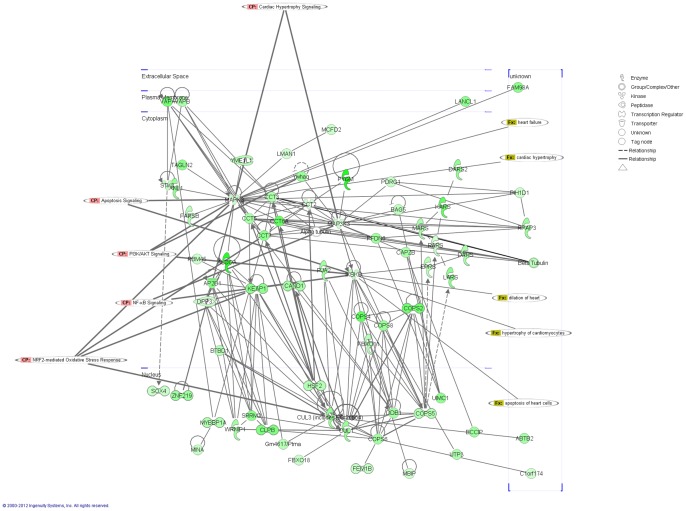
Most significant networks of SDRG associated with cardiac damage generated on the basis of the evidence stored in the IPA library. The top scoring networks identified as “Cardiovascular system development and function; cellular growth and proliferation; post-translational modification” (score 43, focus molecules 35), and “Cell death; cardiovascular system development and function; embryonic development” (score 38, focus molecule 33) were merged and overlaid for cardiovascular disorders.

**Figure 7:**
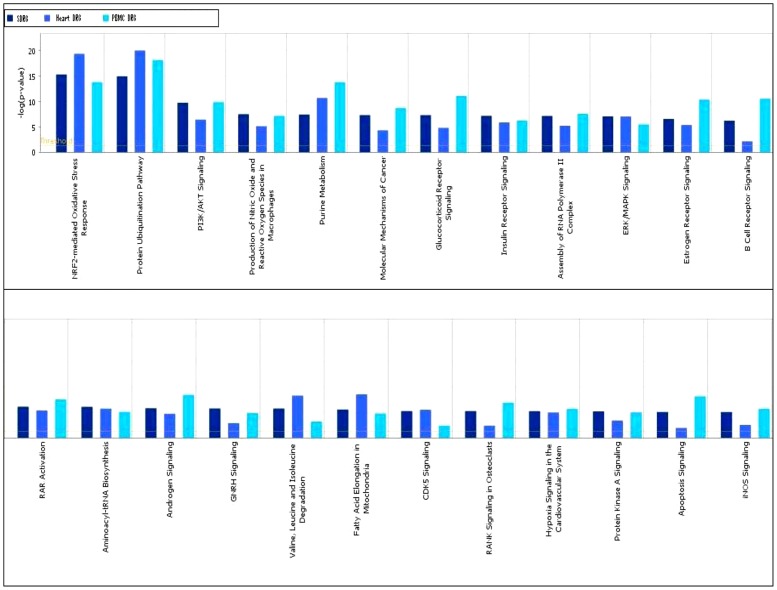
Global Canonical Pathway analysis and comparison of the most significantly enriched canonical pathways that were significantly differentially regulated in the heart vs cont, PBMC vs cont and SDRG datasets. Data sets were analyzed by the IPA software (Ingenuity® Systems, http://www.ingenuity.com). The significance is expressed as a P-value, which was calculated using the right-tailed Fisher's Exact Test. Threshold: P<0.05. The full size figure is available as Figure S1.

IPA was used to identify the biological mechanisms, pathways and functions most relevant to the 2,411 SDRG. The analysis of the SDRG by IPA was composed of 25 networks (Table S3) with the two most significant networks identified as “Cardiovascular system development and function; cellular growth and proliferation; post-translational modification” (score 43, focus molecules 35), and “Cell death; cardiovascular system development and function; embryonic development” (score 38, focus molecule 33). Figure 6 represents an image of the merged networks overlaid for cardiac disorders. The key molecules IKBKB (NFKBIKB, IKKB) [48],[49] and [50] and MAPK3 [51] and [52] were reported to play an important role in cardiac disorders, such as heart failure, heart dilation, hypertrophy and apoptosis. SDRG were also subjected to canonical pathways analysis via IPA software (Fig. 7). The most significantly represented canonical pathways in SDRG were the nuclear factor erythroid 2-related factor (NRF2)-mediated oxidative stress response (59/192 molecules), protein ubiquitination (70/274 molecules) and PI3K/AKT signaling (36/140 molecules). NRF2 and protein ubiquination were also the most significant pathways in DOX-treated hearts vs controls, as well as the treated PBMCs vs controls datasets.

**Figure 8:**
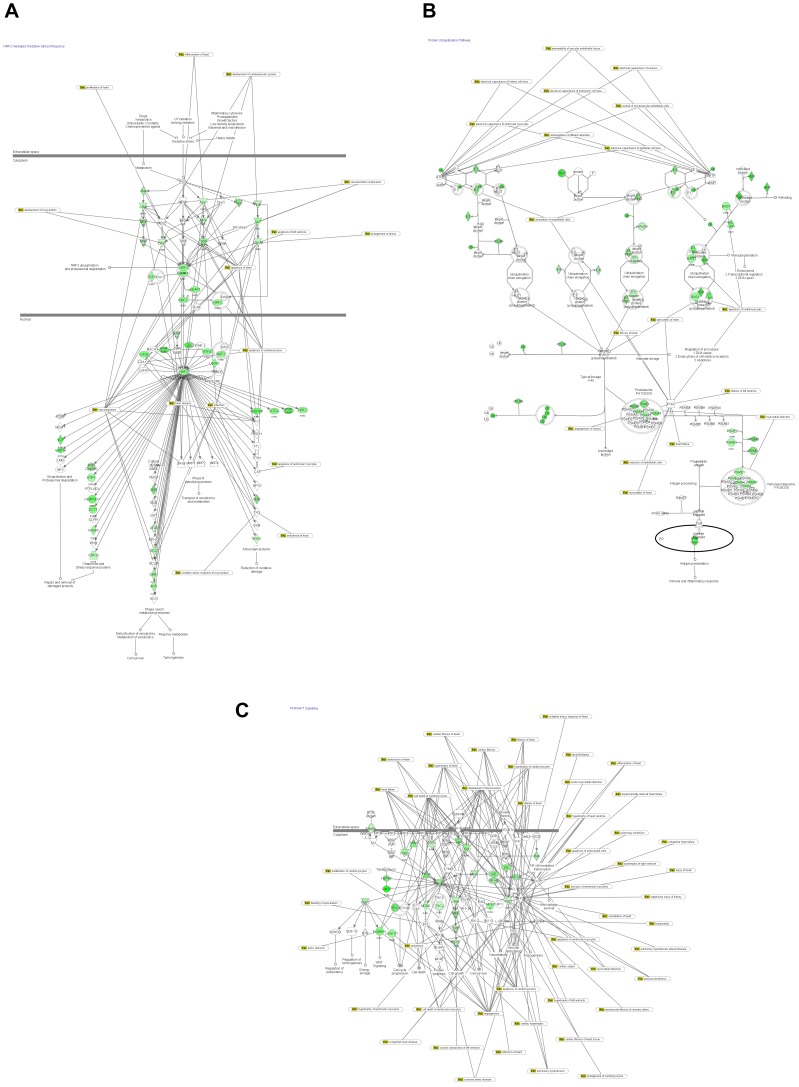
Graphical representation of NRF2-mediated oxidative stress response pathway (A), protein ubiquitination pathway (B) and PI3K/AKT canonical pathways (C), (IPA), overlaid for cardiovascular disease. Green-notes indicate down-regulated gene.

It has been reported that NRF2-mediated oxidative stress response canonical pathway (Fig. 8A) plays an important role in the defense against oxidative stress, cardiovascular disease, inflammation and cancer [53], [54] and [55]. The main function of NRF2 is to activate the antioxidant response and induce transcription of a wide array of cytoprotective genes in response to oxidative stress [56], [57], such as glutamate cysteine ligase (GCL), heme oxygenase-1 (HMOX-1), glutathione S-transferase (GST), NAD(P)H quinine oxidoreductase-1(NQO1), heme oxygenase (HO) and multidrug resistance-associated proteins (MRPs) that are able to combat the harmful effects of oxidative stress, thus restoring intracellular homeostasis. High levels of NRF2 protect cancer cells from the effects of various chemotherapeutic drugs, whereas knockdown of NRF2, transiently or stably, increases the sensitivity of cancer cells to chemotherapeutic-induced cell death [58] In the present study DOX significantly down-regulated the expression of NRF2 (−2.085), GST (−1.923), GCL (−1.776) and HO (−2.849) in the hearts and down-regulated GCLC, GSTM1, GSTM2, GSTM5, GSTK1K1, NFE2L2, CBR1, all of which could be related both to the cardiotoxicity and anti-cancer efficiency of DOX.

Under non-stressful conditions, NRF2 is constantly ubiquitinated and degraded in proteasomes via the ubiquitin proteasome pathway by the CUL3–KEAP1 ubiquitin E3 ligase complex [59]. Protein Ubiquitiination Pathway (Fig. 8B) plays a major role in the degradation of short-lived or regulatory proteins involved in a variety of cellular processes including cell cycle, cell proliferation, apoptosis, DNA repair, transcription regulation, cell surface receptor and ion channel regulation, and antigen presentation [60], [61] and [62]. It has been reported that DOX enhances ubiquitin-proteasome system (UPS)-mediated proteolysis in the heart, suggesting that altered function of the UPS may also be an important mechanism for acute cardiotoxicity of DOX [63]. In the present study, DOX treatment differentially regulated 116 molecules in the hearts and 119 molecules in PBMC associated with this pathway.

The PI3K/AKT pathway signaling plays a central role in apoptosis, inflammation, and cytoskeletal reorganization [64] and [65]. It has been suggested that PI3K-AKT signaling pathway is critically involved in DOX-induced cardiac hypertrophy [66]. In the present study, DOX treatment differentially regulated several genes in the PI3K-AKT pathway which were associated with apoptosis of ventricular myocytes, hypertrophy of ventricular myocytes, systolic dysfunction of the left ventricle and acute myocardial infarction (Fig. 8C).

#### Functional analyses comparison of datasets

**Figure 9:**
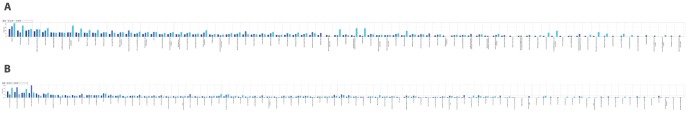
Global functional analysis and comparison of the three datasets. The significance value associated with a function in Global Analysis is a measure of probability that genes from the dataset under investigation participate in that function. (A) Bio-function analysis and comparison; (B) Tox-function analysis and comparison.

The functional analysis and the comparison among the three datasets revealed changes in the biological states across the datasets and helped us to understand cardiomyoctes' and PBMCs' cellular response to DOX in terms of biological processes, clinical pathological end-points, diseases and pathways. Figure 9 shows the comparison of the top 12 bio-functions (Fig. 9A) and top 12 tox-functions (Fig. 9B) of the three datasets. The most significantly affected top bio-function in the SDRG dataset, as well as in the other two datasets was associated with cell death/apoptosis (Table S4). The top significantly affected tox-functions of the three datasets were also associated with cell death/necrosis of the heart, liver and kidneys (Table S5). These results are in agreement with the data presented in recent reviews on DOX-induced cardiotoxicity, which indicate that DOX cardiotoxicity is multifactorial [67], [68] and that cardiomyocyte death by apoptosis and necrosis is a primary mechanism of DOX-induced cardiomyopathy [69].

### Biomarkers of cardiovascular diseases

**Figure 10:**
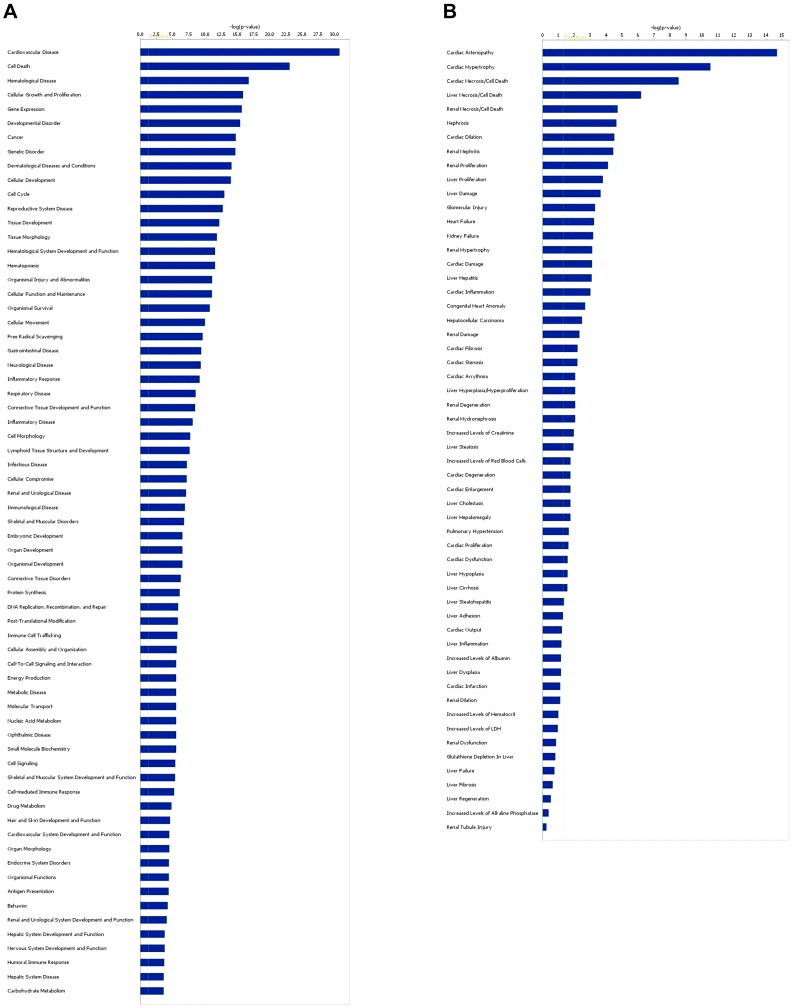
Global functional analysis of the dataset of genes differentially regulated by DOX treatment filtered for cardiovascular diseases. The significance is expressed as a p-value which was calculated using the right-tailed Fisher's Exact Test. (A) Bio-function analysis; (B) Tox-function analysis.

A search in the IPA database for potential biomarkers of cardiovascular disorders and diseases, including cardiomyopathy, heart failure, ischemia, coronary artery disease, hypertension and cardiac hypertrophy within the SDRG dataset produced 188 molecules (Table S6). Figure 10 shows the top bio-function (A) and top tox-function (B) of the biomarker genes filtered from the SDRG gene dataset. Most of the genes within this dataset were reported to affect cardiovascular diseases. Based on the published data, the IPA predicted bio-functions include decreased transcription and expression of RNA; increased hyperplasia, inflammation, connective tissue growth and lipids; and decreased quantity of myeloid cells. IPA predicts a stimulation of cardiovascular disorder development due to the significant down-regulation of the following genes: MDM2 [70], RAF1 [71], HDAC5 [72], NAMPT, [73], PDPK1 [74], MAPK14 [75], JUND [56], TFAM [56], NT5E and DUSP1 [76], SOD1 [77], SOD2 [78], TXN2 [61], MIF [79], PRNP [80], CTNNA1 [81], FHL2 [82], PPP3CA [83], CBR1 [84] and IGF2R [85]. The two up-regulated genes in this dataset, S100A8 and S100A9 have also been associated with cardiovascular diseases. Increased levels of S100A8 (calgranulin A or migration inhibitory factor-related protein 8; MRP-8) and its binding partner S100A9 (calgranulin B, or MRP-14), members of the S100 calcium-binding family of proteins were reported in cardiovascular diseases [86], inflammatory and autoimmune states [87], [88].

### QPCR analysis of selected genes

**Figure 11:**
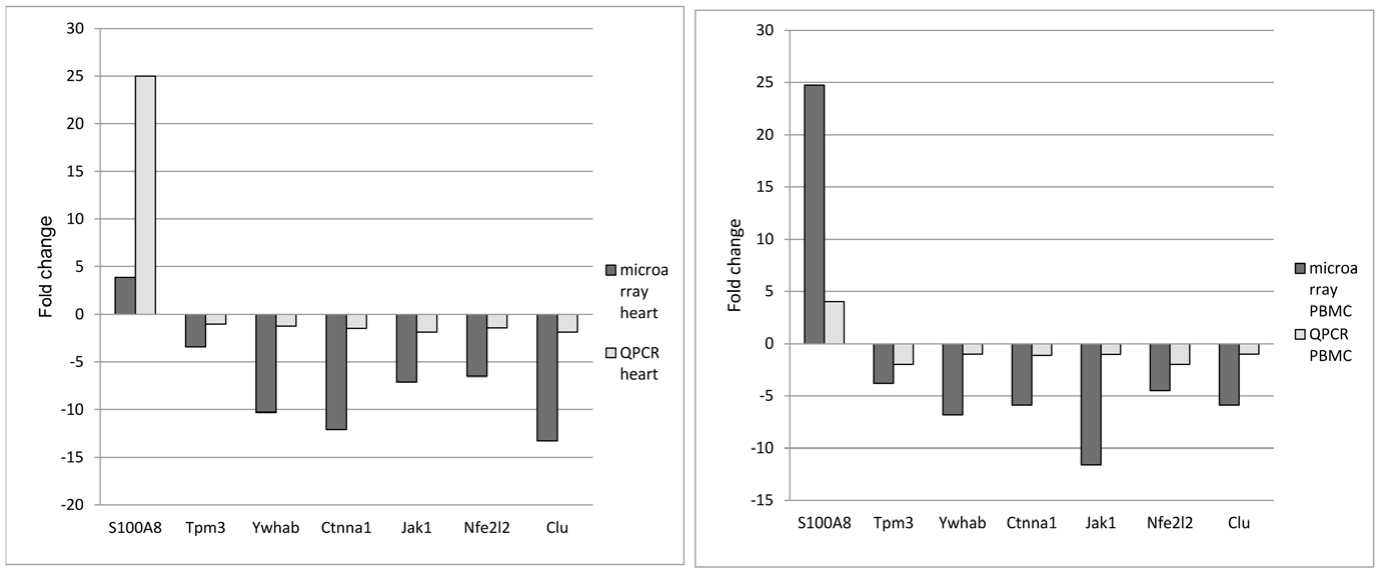
Quantitative real-time PCR validation of 9 genes that were differentially regulated in hearts (A) and PBMC (B) of rats 48 hours after treatment with DOX.

Using QPCR, a comparison between the gene expression regulation of the heart and PBMC was performed in order to confirm the similarities in gene expression detected with gene expression arrays. Several genes were selected for QPCR from the list of SDRG and were amplified. The results presented on Figure 11 confirmed the data from the microarrays.

## Discussion

DOX-based chemotherapy has greatly increased the number of long-term cancer survivals but has also lead to an increasing number of patients experiencing DOX-induced cardiotoxicity. Identification of biomarkers of individual patient sensitivity to the cardiotoxic effects of DOX could improve the safety of chemotherapy.

The present study aimed to determine the feasibility of using the peripheral-blood transcriptome as a potential “surrogate” biomarker of pre-symptomatic detection of DOX-induced cardiotoxicity. We have used a rat model of DOX-induced cardiotoxicity, which resembles the physiological and histological findings in patients [89], [90], [91]. We have analyzed >22,000 transcripts in the hearts and PBMCs of rats 48 hours after a single dose (12 mg/kg) of DOX, similar to the single dose of DOX (often one of several doses over time) used in the treatment of human cancer (65–75 mg/m^2^) [30]. A large number of DRG (45%–48% of the expressed genes being identified as significant, FDR<0.05, fold change >2) were identified in both heart and PBMC. The analysis of heart and PBMC DRG showed their association with multiple pathways, the 5 most significantly affected in the heart being mitochondrial dysfunction, protein ubiquination, NRF2-oxidative stress response, oxidative phosphorylation and EIF2 signaling; and in PBMC they were protein ubiquination, CD28 signaling in T helper cells, T cell receptor signaling, NRF2-mediated oxidative stress response and purine metabolism. The 2,411 SDRG, which were 26.4% of the expressed genes were associated with NRF2-mediated oxidative stress response, protein ubiquination, PI3K/AKT signaling, production of nitric oxide and purine metabolism. Overall, the comparison of the three datasets, heart DRG, PBMC DRG and SDRG showed that oxidative stress-related pathways were key pathways affected by DOX. These results are in agreement with studies showing that a critical mechanism of DOX cardiotoxicity is the free radical-induced oxidative stress and reactive oxygen species (ROS) [92], [93] and [94]. The oxidative damage of tissues that have abundant antioxidant enzymes are usually protected from oxidative damages. The heart however, contains low levels of those enzymes which renders it vulnerable to free radical damage or cardiotoxicity [95]. It has been reported that the level of DOX-induced oxidative stress was found to be up to 10 times greater in the heart than in the liver, kidney or spleen [96] and [97]. Recent reviews [66] and [98] state that DOX-induced cardiomyopathy is likely a result of the summation and mutual feedback of diverse processes such as oxidative stress involving ROS and iron, inhibition of nucleic acid and protein synthesis; release of vasoactive amines; changes in adrenergic function and adenylate cyclase; abnormalities in Ca^2+^ handling; activation of innate immunity, activation of the ubiquitin-proteasome system, downregulation of pro-survival gene (NRG1 and ErB4) expression, and impaired cardiac repair due to inhibition of bone marrow, cardiac progenitor cell, and/or endothelial cell function. It is generally accepted that the mechanisms of antitumor action of DOX are distinct from the mechanisms of its cardiotoxicity. The mechanisms of antitumor activity are thought to be due to the DNA damage and inhibition of cell replication of highly proliferative tumors that include intercalation into DNA, and interference with DNA unwinding or DNA strand separation via inhibition of topoisomerase II, all of which result in apoptosis [99] and [100]. Since adult cardiomyocytes are terminally differentiated with minimally replicative capacity they should be resistant to the antimitotic mechanisms of DOX [101].

The analysis of the SDRG dataset in the present study using IPA software identified a set of 188 genes potentially indicative of cardiovascular disease. Most of these genes were reported to affect cardiovascular diseases via decreased transcription and expression of RNA; increased hyperplasia, inflammation and growth of connective tissue cells; increased quantity of lipids and decreased quantity of myeloid cells. Vanburen et al [102] identified a blood cell gene expression signature consisting of 197 “mortality genes” associated with chronic heart failure (CHF) in 71 patients. Several of the genes reported in their study were in fact similarly differentially downregulated in our present study, including CALM1 (calmodulin 1), PRKCA (Protein kinase C, alpha), PRKCB (Protein kinase C, beta) and PRKCH (Protein kinase C). A study by Blanco et al. [84] found that single polymorphism in the CBR3 gene was associated with a dose-dependent risk of anthracycline-related cardiomyopathy in childhood cancer survivors. A recent review on S100A8 and S100A9 involvement in cardiovascular biology and disease indicate that increased plasma levels of S100A8/A9 predict cardiovascular events in humans, and deletion of these proteins partly protects mice from atherosclerosis [103]. Increased plasma levels of S100A9 was a predictor for future nonfatal myocardial infarction among healthy women [104] and S100A8/A9 heterocomplex was found to be an early marker for detection of acute coronary syndrome [105]. Studies in mice indicate that S100A8/9 might contribute to cardiovascular dysfunction resulting from sepsis [106] and atherosclerosis [107]. Using a cDNA array with 13,000 features representing more than 5,000 genes cloned from skeletal and cardiac muscle Yi et al. [108] determined the cardiac gene expression profiles of 2 mouse models of DOX-induced cardiomyopathy: an acute model in which the mice were treated with a single injection of 15 mg/kg DOX and a chronic model, in which the mice received 3 mg/kg DOX weekly for 12 weeks. The acute model showed a greater number of dysregulated genes (90 genes) than did the chronic model (23 genes) and most of the transcripts rapidly reverted to baseline with a time course similar to the half-life clearance of the drug. Most of the regulated genes in both models fall into similar functional categories: 1) metabolism and oxidative stress response proteins; 2) signal transduction proteins; 3) apoptotic factors; and 4) cardiac muscle structural proteins. Berthiaume and Wallace [109] determined the transcription profile of late DOX-induced cardiotoxicity in rats and showed that a major element of persistent DOX cardiotoxicity is the modulated expression of mitochondria-related transcripts. The present study has provided a global *in vivo* expression profiling and pathway analysis of early DOX-induced gene perturbation in the hearts and PBMC induced by DOX. It has generated comprehensive information on the biological mechanisms that are related to DOX cardiotoxicity and confirmed the data accumulated by multiple studies that DOX cardiotoxicity is multifactorial with oxidative stress playing a predominant role. We have identified a large number of SDRG associated with multiple pathways and a group 188 SDRG potentially indicative of cardiovascular disease.

### Conclusion

In conclusion, this study provides important information about the feasibility of using the PBMC as a surrogate marker for DOX-induced cardiotoxicity. We have identified a PBMC transcriptome signature induced by a single dose of DOX similar to the single dose administered in humans, indicating that blood cell gene-expression profiling may serve as a biomarker for early detection of DOX-induced cardiotoxicity. The data obtained lay a foundation for future clinical studies to analyze the gene expression of cancer patients treated with DOX-based chemotherapy. Significantly, the analysis of the differential gene expression in PBMC might reveal indicators of subtle injury to the heart induced by the initial, low dose of DOX which does not result in clinically sufficient toxicity as defined by the methods currently used in oncologic practices.

## Supporting Information

Figure S1 **Full version of** **Figure 7** **.**
(TIF)

Table S1 **Genome-wide expression profiling datasets.** Statistical significance was set at FDR<0.05. Only statistically significant genes with FC>2 in expression between groups were retained.
(XLSX)

Table S2 **Gene ontology analysis of SDRG.** The most significantly enriched categories (FDR<0.05) are enlisted.
(XLSX)

Table S3 **SDRG networks identified by IPA.**
(XLS)

Table S4 **IPA identified significantly affected bio-function in the SDRG dataset.**
(XLS)

Table S5 **IPA identified significantly affected tox-functions in the SDRG dataset.**
(XLS)

Table S6 **Potential biomarkers of cardiovascular disorders and diseases within the SDRG dataset identified by IPA.**
(XLS)

## References

[1] Smith LA, Cornelius VR, Plummer CJ, Levitt G, Verrill M, Canney P, Jones A (2010) Cardiotoxicity of anthracycline agents for the treatment of cancer: systematic review and meta-analysis of randomised controlled trials. BMC Cancer 10: 337–345. 20587042 10.1186/1471-2407-10-337 PMC2907344

[2] Gharib MI, Burnett AK (2002) Chemotherapy-induced cardiotoxicity: current practice and prospects of prophylaxis. Eur J Heart Fail 4: 235–42. 12034146 10.1016/s1388-9842(01)00201-x

[3] Floyd JD, Nguyen DT, Lobins RL, Bashir Q, Doll DC, et al (2005) Cardiotoxicity of cancer therapy. J Clin Oncol 23: 7685–94. 16234530 10.1200/JCO.2005.08.789

[4] Singal PK, Iliskovic N (1998) Doxorubicin-induced cardiomyopathy. N Engl J Med 339: 900–912. 9744975 10.1056/NEJM199809243391307

[5] Swain SM, Whaley FS, Ewer MS (2003) Congestive heart failure in patients treated with doxorubicin: a retrospective analysis of three trials. Cancer 97: 2869–79. 12767102 10.1002/cncr.11407

[6] Kremer LC, van der Pal HJ, Offringa M, van Dalen EC, Voûte PA (2002) Frequency and risk factors of subclinical cardiotoxicity after anthracycline therapy in children: a systematic review. Ann Oncol 13: 819–29. 12123328 10.1093/annonc/mdf167

[7] Shan K, Lincoff AM, Young JB (1996) Anthracycline-induced cardiotoxicity. Ann Intern Med 125: 47–58. 8644988 10.7326/0003-4819-125-1-199607010-00008

[8] Giantris A, Abdurrahman L, Hinkle A, Asselin B, Lipshultz SE (1998) Anthracycline-induced cardiotoxicity in children and young adults. Crit Rev Oncol Hematol 27: 53–68. 9548017 10.1016/s1040-8428(97)10007-5

[9] Wexler LH, Andrich MP, Venzon D, Berg SL, Weaver-McClure L, et al (1996) Randomized trial of the cardioprotective agent ICRF-187 in pediatric sarcoma patients treated with doxorubicin. J Clin Oncol 14: 362–372. 8636745 10.1200/JCO.1996.14.2.362

[10] Bovelli D, Plataniotis G, Roila F (2010) ESMO Guidelines Working Group. Cardiotoxicity of chemotherapeutic agents and radiotherapy-related heart disease. ESMO Clinical Practice Guidelines. Ann Oncol 21: Suppl 5: v277–82. 20555097 10.1093/annonc/mdq200

[11] Menna P, Gonzalez Paz O, Chello M, Covino E, Salvatorelli E, et al (2012) Anthracycline cardiotoxicity. Expert Opin Drug Saf 11: Suppl 1, S21–36. 10.1517/14740338.2011.589834 21635149

[12] Barry E, Alvarez JA, Scully RE, Miller TL, Lipshultz SE (2007) Anthracycline-induced cardiotoxicity: course, pathophysiology, prevention and management. Expert Opin Pharmacother 8: 1039–58. 17516870 10.1517/14656566.8.8.1039

[13] Gianni L, Herman EH, Lipshultz SE, Minotti G, Sarvazyan N, et al (2008) Anthracycline cardiotoxicity: from bench to bedside. J Clin Oncol 26: 3777–84. 18669466 10.1200/JCO.2007.14.9401 PMC3018290

[14] Jain D (2000) Cardiotoxicity of doxorubicin and other anthracycline derivatives. J Nucl Cardiol 7: 53–68. 10698235 10.1067/mnc.2000.103324

[15] Swain SM, Whaley FS, Ewer MS (2003) Congestive heart failure in patients treated with doxorubicin: a retrospective analysis of three trials. Cancer 97: 2869. 12767102 10.1002/cncr.11407

[16] Pai VB, Nahata MC (2000) Cardiotoxicity of chemotherapeutic agents: incidence, treatment and prevention. Drug Saf 22: 263–302. 10789823 10.2165/00002018-200022040-00002

[17] Jaffe AS, Ravkilde J, Roberts R, Naslund U, Apple FS, et al (2000) It's time for a change to a troponin standard. Circulation 102: 1216–20. 10982533 10.1161/01.cir.102.11.1216

[18] Yasue H, Yoshimura M, Sumida H, Kikuta K, Kagiyama K, et al (1994) Localization and mechanism of secretion of B-type natriuretic peptide in comparison with those of A-type natriuretic peptide in normal subjects and patients with heart failure. Circulation 90: 195–203. 8025996 10.1161/01.cir.90.1.195

[19] Herman EH, Lipshultz SE, Ferrans VJ (2003) The use of cardiac biomarkers to detect myocardial damage induced by chemotherapeutic agents. In: Wu AHB, ed. Cardiac Markers. 2nd ed. Totowa, NJ: Humana Press, 87–109

[20] Dolci A, Dominici R, Cardinale D, Sandri MT, Panteghini M (2008) Biochemical markers for prediction of chemotherapy-induced cardiotoxicity: systematic review of the literature and recommendations for use. Am J Clin Pathol 130: 688–710. 18854260 10.1309/AJCPB66LRIIVMQDR

[21] Lu P (2005) Monitoring cardiac function in patients receiving doxorubicin. Semin Nucl Med 35: 197–201. 16098293 10.1053/j.semnuclmed.2005.02.005

[22] Swain SM, Whaley FS, Ewer MS (2003) Congestive heart failure in patients treated with doxorubicin: a retrospective analysis of three trials. Cancer 97: 2869–79. 12767102 10.1002/cncr.11407

[23] Elliott P (2006) Pathogenesis of cardiotoxicity induced by anthracyclines. Semin Oncol 33: 3 Suppl 8 S2–7. 10.1053/j.seminoncol.2006.04.020 16781283

[24] Wouters KA, Kremer LCM, Miller TL, Herman EH, Lipshultz SE (2005) Protecting against anthracycline-induced myocardial damage: a review of the most promising strategies. Br J Haematol 131: 561–78. 16351632 10.1111/j.1365-2141.2005.05759.x

[25] Staratschek-Jox A, Classen S, Gaarz A, Debey-Pascher S, Schultze JL Blood-based transcriptomics: Leukemias and beyond. Expert Rev Mol Diagn 9 2009 271–280. 19379085 10.1586/erm.09.9

[26] Mohr S, Liew CC (2007) The peripheral-blood transcriptome: new insights into disease and risk assessment. Trends Mol Med 13: 422. 17919976 10.1016/j.molmed.2007.08.003

[27] Umbright C, Sellamuth R, Li S, Kashon M, Luster M, et al (2010) Blood gene expression markers to detect and distinguish target organ toxicity. Mol Cell Biochem 335: 223–234. 19784758 10.1007/s11010-009-0272-5

[28] Liew CC, Ma J, Tang HC, Zheng R, Dempsey AA (2006) The peripheral blood transcriptome dynamically reflects system wide biology: a potential diagnostic tool. J Lab Clin Med 147: 126–130. 16503242 10.1016/j.lab.2005.10.005

[29] Ikeda Y, Aihara K, Akaike M, Sato T, Ishikawa K, et al (2010) Androgen receptor counteracts Doxorubicin-induced cardiotoxicity in male mice. Mol Endocrinol 24: 1338–48. 20501642 10.1210/me.2009-0402 PMC5417461

[30] Guidance for industry estimating the maximum safe starting dose in initial clinical trials for therapeutics in adult healthy volunteers (2005) U.S. Department of Health and Human Services. Food and Drug Administration. Center for Drug Evaluation and Research (CDER) July 2005 Pharmacology and Toxicology.

[31] Hayward R, Hydock DS (2007) Doxorubicin cardiotoxicity in the rat: an in vivo characterization. J Am Assoc Lab Anim Sci 46: 20–32. 17645292

[32] Medicine and Healthcare products Regulatory Agency website. Available: http://www.mhra.gov.uk/home/groups/l-unit1/documents/websiteresources/con2024436.pdf. Accessed 2012 May 20.

[33] Todorova VK, Kaufmann Y, Hennings LJ, Klimberg VS (2010) Glutamine regulation of doxorubicin accumulation in hearts versus tumors in experimental rats. Cancer Chem Pharmacol 66: 315–23. 10.1007/s00280-009-1165-8 19898822

[34] Zhao Y, Wang H, Gustafsson M, Muraro A, Bruhn S, et al (2012) Combined multivariate and pathway analyses show that allergen-induced gene expression changes in CD4+ T cells are reversed by glucocorticoids. PLoS One 7: e39016. 22701743 10.1371/journal.pone.0039016 PMC3373548

[35] Wright WR, Parzych K, Crawford D, Mein C, Mitchell JA, Paul-Clark MJ (2012) Inflammatory transcriptome profiling of human monocytes exposed acutely to cigarette smoke. PLoS One 7: e30120. 22363418 10.1371/journal.pone.0030120 PMC3281820

[36] Allison DB, Cui X, Page GP, Sabripour M (2006) Microarray data analysis: from disarray to consolidation and consensus. Nat Rev Genet 7: 55–65. 16369572 10.1038/nrg1749

[37] Shmookler Reis RJ, Ayyadevara S, Crow WA, Lee T, Delongchamp RR (2012) Gene categories differentially expressed in C. elegans age-1 mutants of extraordinary longevity: New insights from novel data-mining procedures. J Gerontol A Biol Sci Med Sci 67: 366–75. 22021389 10.1093/gerona/glr186

[38] Delongchamp RR, Velasco C, Dial S, Harris A (2005) Genome-wide estimation of gender differences in the expression of human liver genes: Statistical design and analysis. BMC Bioinformatics 6: S13. 10.1186/1471-2105-6-S2-S13 PMC1637036 16026598

[39] Wright GW, Simon RM (2003) A random variance model for detection of differential gene expression in small microarray experiments. Bioinformatics 19: 2448–55. 14668230 10.1093/bioinformatics/btg345

[40] Efron B (2004) Large-Scale Simultaneous Hypothesis Testing: The Choice of a Null Hypothesis. JASA 99: 96–104.

[41] Storey JD (2002) A direct approach to false discovery rates. J Roy Stat Soc B 64: 479–98.

[42] Delongchamp R, Lee T, Velasco C (2006) A method for computing the overall statistical significance of a treatment effect among a group of genes. BMC Bioinformatics 7: Suppl2 S11. 10.1186/1471-2105-7-S2-S11 PMC1683577 17118132

[43] Lee T, Desai VG, Velasco C, Shmookler Reis RJ, Delongchamp RR (2008) Testing for treatment effects on gene ontology. BMC Bioinformatics 9: Suppl 9, S20. 10.1186/1471-2105-9-S9-S20 PMC2537571 18793466

[44] Benjamini Y, Hochberg Y (1995) Controlling the false discovery rate: a practical and powerful approach to multiple testing. Journal of the Royal Statistical Society B 57: 289–300.

[45] Livak KJ, Schmittgen TD (2001) Analysis of relative gene expression data using real-time quantitative PCR and the 2(−Delta Delta C(T)) Method. Methods 25: 402–408. 11846609 10.1006/meth.2001.1262

[46] Delongchamp RR, Bowyer JF, Chen JJ, Kodell RL (2004) Multiple-testing strategy for analyzing cDNA array data on gene expression. Biometrics 60: 774–82. 15339301 10.1111/j.0006-341X.2004.00228.x

[47] Schweder T, Spjotvoll E (1982) Plots of p-values to evaluate many tests simultaneously. Biometrika 69: 493–502.

[48] Islam KN, Koch WJ (2012) Involvement of nuclear factor κB (NF-κB) signaling pathway in regulation of cardiac G protein-coupled receptor kinase 5 (GRK5) expression. J Biol Chem 287: 12771–8. 22389501 10.1074/jbc.M111.324566 PMC3339976

[49] Purcell NH, Tang G, Yu C, Mercurio F, DiDonato JA, et al (2001) Activation of NF-kappa B is required for hypertrophic growth of primary rat neonatal ventricular cardiomyocytes. Proc Natl Acad Sci U S A 98: 6668–73. 11381115 10.1073/pnas.111155798 PMC34410

[50] Hikoso S, Yamaguchi O, Nakano Y, Takeda T, Omiya S, et al (2009) The I{kappa}B kinase {beta}/nuclear factor {kappa}B signaling pathway protects the heart from hemodynamic stress mediated by the regulation of manganese superoxide dismutase expression. Circ Res 105: 70–9. 19478205 10.1161/CIRCRESAHA.108.193318

[51] Purcell NH, Wilkins BJ, York A, Saba-El-Leil MK, Meloche S, et al (2007) Genetic inhibition of cardiac ERK1/2 promotes stress-induced apoptosis and heart failure but has no effect on hypertrophy in vivo. Proc Natl Acad Sci USA 104: 14074–9. 17709754 10.1073/pnas.0610906104 PMC1955824

[52] Golden HB, Watson LE, Lal H, Verma SK, Foster DM, et al (2009) Anthrax toxin: pathologic effects on the cardiovascular system. Front Biosci 14: 2335–57. 10.2741/3382 19273204

[53] Nguyen T, Nioi P, Pickett CB (2009) The Nrf2-antioxidant response element signaling pathway and its activation by oxidative stress. J Biol Chem 284: 13291–5. 19182219 10.1074/jbc.R900010200 PMC2679427

[54] Zhang DD (2006) Mechanistic studies of the Nrf2-Keap1 signaling pathway. Drug Metab Rev 38: 769–789. 17145701 10.1080/03602530600971974

[55] Motohashi H, Yamamoto M (2004) Nrf2-Keap1 defines a physiologically important stress response mechanism. Trends Mol Med 10: 549–557. 15519281 10.1016/j.molmed.2004.09.003

[56] Hayes JD, McMahon M (2001) Molecular basis for the contribution of the antioxidant responsive element to cancer chemoprevention. Cancer Lett 174: 103–113. 11689285 10.1016/s0304-3835(01)00695-4

[57] Kensler TW, Wakabayashi N, Biswal S (2007) Cell survival responses to environmental stresses via the Keap1-Nrf2-ARE pathway. Annu Rev Pharmacol Toxicol 47: 89–116. 16968214 10.1146/annurev.pharmtox.46.120604.141046

[58] Wang XJ, Sun Z, Villeneuve NF, Zhang S, Zhao F, et al (2002) Nrf2 enhances resistance of cancer cells to chemotherapeutic drugs, the dark side of Nrf2. Carcinogenesis 29: 1235–1243. 10.1093/carcin/bgn095 PMC3312612 18413364

[59] Sekhar KR, Yan XX, Freeman L (2002) Nrf2 degradation by the ubiquitin proteasome pathway is inhibited by KIAA0132, the human homolog to INrf2. Oncogene 21: 6829–6834. 12360409 10.1038/sj.onc.1205905

[60] Jesenberger V, Jentsch S (2002) Deadly encounter: ubiquitin meets apoptosis. Nat Rev Mol Cell Biol 3: 112–21. 11836513 10.1038/nrm731

[61] Glickman MH, Ciechanover A (2002) The ubiquitin-proteasome proteolytic pathway: destruction for the sake of construction. Physiol Rev 82: 373–428. 11917093 10.1152/physrev.00027.2001

[62] Wójcik C (2002) Regulation of apoptosis by the ubiquitin and proteasome pathway. J Cell Mol Med 6: 25–48. 12003667 10.1111/j.1582-4934.2002.tb00309.x PMC6740140

[63] Kumarapeli AR, Horak KM, Glasford JW, Li J, Chen Q, et al (2005) A novel transgenic mouse model reveals deregulation of the ubiquitin-proteasome system in the heart by doxorubicin. FASEB J 19: 2051–3. 16188962 10.1096/fj.05-3973fje

[64] Deane JA, Fruman DA (2004) Phosphoinositide 3-kinase: diverse roles in immune cell activation. Annu Rev Immunol 22: 563–598. 15032589 10.1146/annurev.immunol.22.012703.104721

[65] Underhill DM, Ozinsky A (2002) Phagocytosis of microbes: complexity in action. Annu. Rev Immunol 20: 825–852. 10.1146/annurev.immunol.20.103001.114744 11861619

[66] Merten KE, Jiang Y, Feng W, Kang YJ (2006) Calcineurin activation is not necessary for Doxorubicin-induced hypertrophy in H9c2 embryonic rat cardiac cells: involvement of the phosphoinositide 3-kinase-Akt pathway. J Pharmacol Exp Ther 319: 934–40. 16926266 10.1124/jpet.106.108845

[67] Wei JY (1995) Cardiovascular comorbidity in the older cancer patients. Seminars in Oncol 22: 9–10. 7863352

[68] Minotti G, Menna P, Salvatorelli E, Cairo G, Gianni L (2004) Anthracyclines: molecular advances and pharmacologic developments in antitumor activity and cardiotoxicity. Pharmacol Rev 56: 185–229. 15169927 10.1124/pr.56.2.6

[69] Zhang YW, Shi J, Li YJ, Wei L (2009) Cardiomyocyte death in doxorubicin-induced cardiotoxicity. Arch Immunol Ther Exp (Warsz) 57: 435–45. 19866340 10.1007/s00005-009-0051-8 PMC2809808

[70] Toth A, Nickson P, Qin LL, Erhardt P (2006) Differential Regulation of Cardiomyocyte Survival and Hypertrophy by MDM2, an E3 Ubiquitin Ligase. J Biol Chem 281: 3679–89. 16339144 10.1074/jbc.M509630200

[71] Golden HB, Watson LE, Lal H, Verma SK, Foster DM, et al (2009) Anthrax toxin: pathologic effects on the cardiovascular system. Front Biosci 14: 2335–57. 10.2741/3382 19273204

[72] Zhang CL, McKinsey TA, Chang S, Antos CL, Hill JA, et al (2002) Class II histone deacetylases act as signal-responsive repressors of cardiac hypertrophy. Cell 110: 479–88. 12202037 10.1016/s0092-8674(02)00861-9 PMC4459650

[73] Wellcome Trust Case Control Consortium (2007) Genome-wide association study of 14,000 cases of seven common diseases and 3,000 shared controls. Nature 447: 661–78. 17554300 10.1038/nature05911 PMC2719288

[74] Mora A, Davies AM, Bertrand L, Sharif I, Budas GR, et al (2003) Deficiency of PDK1 in cardiac muscle results in heart failure and increased sensitivity to hypoxia. EMBO J 22: 4666–76. 12970179 10.1093/emboj/cdg469 PMC212735

[75] Braz JC, Bueno OF, Liang Q, Wilkins BJ, Dai YS, et al (2003) Targeted inhibition of p38 MAPK promotes hypertrophic cardiomyopathy through upregulation of calcineurin-NFAT signaling. J Clin Invest 111: 1475–86. 12750397 10.1172/JCI17295 PMC155046

[76] Bueno OF, De Windt LJ, Lim HW, Tymitz KM, Witt SA, et al (2001) The dual-specificity phosphatase MKP-1 limits the cardiac hypertrophic response in vitro and in vivo. Circ Res 88: 88–96. 11139479 10.1161/01.res.88.1.88

[77] Infanger DW, Sharma RV, Davisson RL (2006) NADPH oxidases of the brain: distribution, regulation, and function. Antioxid Redox Signal 8: 1583–96. 16987013 10.1089/ars.2006.8.1583

[78] Widder JD, Fraccarollo D, Galuppo P, Hansen JM, Jones DP, et al (2009) Attenuation of angiotensin II-induced vascular dysfunction and hypertension by overexpression of Thioredoxin. Hypertension 54: 338–44. 19506101 10.1161/HYPERTENSIONAHA.108.127928 PMC2752391

[79] Li H, Gao Y, Qi Y, Katovich MJ, Jiang N, et al (2008) Macrophage migration inhibitory factor in hypothalamic paraventricular nucleus neurons decreases blood pressure in spontaneously hypertensive rats. FASEB J 22 2008 3175–85. 18535252 10.1096/fj.08-108662 PMC2518251

[80] Wang Y (2007) Mitogen-activated protein kinases in heart development and diseases. Circulation 116: 1413–23. 17875982 10.1161/CIRCULATIONAHA.106.679589 PMC3808829

[81] Sheikh F, Chen Y, Chen Y, Liang X, Hirschy A, et al (2006) alpha-E-catenin inactivation disrupts the cardiomyocyte adherens junction, resulting in cardiomyopathy and susceptibility to wall rupture. Circulation 114: 1046–55. 16923756 10.1161/CIRCULATIONAHA.106.634469

[82] Purcell NH, Darwis D, Bueno OF, Müller JM, Schüle R, et al (2004) Extracellular signal-regulated kinase 2 interacts with and is negatively regulated by the LIM-only protein FHL2 in cardiomyocytes. Mol Cell Biol 24: 1081–95. 14729955 10.1128/MCB.24.3.1081-1095.2004 PMC321437

[83] Gooch JL (2006) An emerging role for calcineurin Aalpha in the development and function of the kidney. Am J Physiol Renal Physiol 290: F769–76. 16527922 10.1152/ajprenal.00281.2005

[84] Blanco JG, Sun CL, Landier W, Chen L, Esparza-Duran D, et al (2012) Anthracycline-related cardiomyopathy after childhood cancer: role of polymorphisms in carbonyl reductase genes–a report from the Children's Oncology Group. J Clin Oncol 30: 1415–21. 22124095 10.1200/JCO.2011.34.8987 PMC3383117

[85] Ghosh P, Dahms NM, Kornfeld S (2003) Mannose 6-phosphate receptors: new twists in the tale. Nat Rev Mol Cell Biol 4: 202–12. 12612639 10.1038/nrm1050

[86] Croce K (2010) S100A8/A9 complex: more than just a biomarker of cardiovascular risk? Circ J 74: 626–627. 20234100 10.1253/circj.cj-10-0192

[87] Perera C, McNeil HP, Geczy CL (2010) S100 Calgranulins in inflammatory arthritis. Immunol Cell Biolm 88: 41–49. 10.1038/icb.2009.88 19935766

[88] Nacken W, Roth J, Sorg C, Kerkhoff C (2003) S100A9: a myeloid S100 representative as a prominent player in innate immunity. Microsc Res Tech 60: 569–580. 12645005 10.1002/jemt.10299

[89] Todorova VK, Kaufmann Y, Hennings LJ, Klimberg VS (2010) Glutamine regulation of doxorubicin accumulation in hearts versus tumors in experimental rats. Cancer Chem Pharmacol 66: 315–23. 10.1007/s00280-009-1165-8 19898822

[90] Todorova V, Kaufmann Y, Hennings L, Klimberg S (2009) Protective effects of glutamine against acute doxorubicin cardiotoxicity in tumor-bearing rats. J Nutr 140: 44–8. 19889810 10.3945/jn.109.113415

[91] Herman EH (1998) Ferrans VJ (1998) Preclinical animal models of cardiac protection from anthracycline-induced cardiotoxicity. Semin Oncol 25: 15–21. 9768819

[92] Iarussi D, Indolfi P, Casale F, Coppolino P, Tedesco MA, et al (2001) Recent advances in the prevention of anthracycline cardiotoxicity in childhood. Curr Med Chem 8: 1649–60. 11562284 10.2174/0929867013371888

[93] Wallace KB (2003) Doxorubicin-induced cardiac mitochondrionopathy. Pharmacol Toxicol 93: 105–15. 12969434 10.1034/j.1600-0773.2003.930301.x

[94] Shi Y, Moon M, Dawood S, McManus B, Liu PP (2011) Mechanisms and management of doxorubicin cardiotoxicity. Herz 36: 296–305. 21656050 10.1007/s00059-011-3470-3

[95] Harvey RA, Champe PC (eds) (1992) Anticancer drugs, in Lippincott's Illustrated Reviews: Pharmacology. Philadelphia, JB Lippincott Company.

[96] Hershman DL, Shao T (2009) Anthracycline cardiotoxicity after breast cancer treatment. Oncology 23: 1–15. 19418823

[97] Singal PK, Iliskovic N (1998) Doxorubicin-induced cardiomyopathy. N Engl J Med 339: 900–905. 9744975 10.1056/NEJM199809243391307

[98] Shi Y, Moon M, Dawood S, McManus B, Liu PP (2011) Mechanisms and management of doxorubicin cardiotoxicity. Herz 36: 296–305. 21656050 10.1007/s00059-011-3470-3

[99] Yi X, Bekeredjian R, DeFilippis NJ, Siddiquee Z, Fernandez E, et al (2006) Transcriptional analysis of doxorubicin-induced cardiotoxicity. Am J Physiol Heart Circ Physiol 290: H1098–102. 16243910 10.1152/ajpheart.00832.2005

[100] Minotti G, Menna P, Salvatorelli E, Cairo G, Gianni L (2004) Anthracyclines: molecular advances and pharmacologic developments in antitumor activity and cardiotoxicity. Pharmacol Rev 56: 185–229. 15169927 10.1124/pr.56.2.6

[101] Kresh JY, Chopra A (2011) Intercellular and extracellular mechanotransduction in cardiac myocytes. Pflugers Arch 462: 75–87. 21437600 10.1007/s00424-011-0954-1

[102] Vanburen P, Ma J, Chao S, Mueller E, Schneider DJ, et al (2011) Blood gene expression signatures associate with heart failure outcomes. Physiol Genomics 43: 392–7. 21266504 10.1152/physiolgenomics.00175.2010 PMC3092336

[103] Averill MM, Kerkhoff C, Bornfeldt KE (2012) S100A8 and S100A9 in cardiovascular biology and disease. Arterioscler Thromb Vasc Biol 32: 223–9. 22095980 10.1161/ATVBAHA.111.236927 PMC3262097

[104] Healy AM, Pickard MD, Pradhan AD, Wang Y, Chen Z, et al (2006) Platelet expression profiling and clinical validation of myeloid-related protein-14 as a novel determinant of cardiovascular events. Circulation 113: 2278–2284. 16682612 10.1161/CIRCULATIONAHA.105.607333

[105] Altwegg LA, Neidhart M, Hersberger M, Müller S, Eberli FR, et al (2007) Myeloid-related protein 8/14 complex is released by monocytes and granulocytes at the site of coronary occlusion: a novel, early, and sensitive marker of acute coronary syndromes. Eur Heart J 28: 941–948. 17387139 10.1093/eurheartj/ehm078

[106] Boyd JH, Kan B, Roberts H, Wang Y, Walley KR (2008) S100A8 and S100A9 mediate endotoxin-induced cardiomyocyte dysfunction via the receptor for advanced glycation end products. Circ Res 102: 1239–1246. 18403730 10.1161/CIRCRESAHA.107.167544

[107] Croce K, Gao H, Wang Y, Mooroka T, Sakuma M, et al (2009) Myeloid-related protein-8/14 is critical for the biological response to vascular injury. Circulation 120: 427–436. 19620505 10.1161/CIRCULATIONAHA.108.814582 PMC3070397

[108] Yi X, Bekeredjian R, DeFilippis NJ, Siddiquee Z, Fernandez E, et al (2006) Transcriptional analysis of doxorubicin-induced cardiotoxicity. Am J Physiol Heart Circ Physiol 290: H1098–102. 16243910 10.1152/ajpheart.00832.2005

[109] Berthiaume JM, Wallace KB (2007) Persistent alterations to the gene expression profile of the heart subsequent to chronic Doxorubicin treatment. Cardiovasc Toxicol 7: 178–86. 17901561 10.1007/s12012-007-0026-0

