# Correction: Transcriptome Profiling of Peripheral Blood Cells Identifies Potential Biomarkers for Doxorubicin Cardiotoxicity in a Rat Model

**DOI:** 10.1371/annotation/843bb7f2-2891-494c-969b-7e30eed37230

**Published:** 2013-02-21

**Authors:** Valentina K. Todorova, Marjorie L. Beggs, Robert R. Delongchamp, Ishwori Dhakal, Issam Makhoul, Jeanne Y. Wei, V. Suzanne Klimberg

The following information was missing from the Funding section: NIH grant (#AG028718) with a pilot project grant to VKT.

